# Trust in embedding co-design for innovation and change: considering the role of senior leaders and managers

**DOI:** 10.1108/JHOM-07-2023-0207

**Published:** 2024-02-21

**Authors:** Tina Bedenik, Claudine Kearney, Éidín Ní Shé

**Affiliations:** School of Population Health, Royal College of Surgeons in Ireland, Dublin, Ireland; Graduate School of Healthcare Management, Royal College of Surgeons in Ireland, Dublin, Ireland

**Keywords:** Co-design, Health systems, Trust, Healthcare innovation, Health managers, Leaders, Embeddedness

## Abstract

**Purpose:**

In this viewpoint article, the authors recognize the increased focus in health systems on co-design for innovation and change. This article explores the role of leaders and mangers in developing and enhancing a culture of trust in their organizations to enable co-design, with the potential to drive innovation and change in healthcare.

**Design/methodology/approach:**

Using social science analyses, the authors argue that current co-design literature has limited focus on interactions between senior leaders and managers, and healthcare staff and service users in supporting co-designed innovation and change. The authors draw on social and health science studies of trust to highlight how the value-based co-design process needs to be supported and enhanced. We outline what co-design innovation and change involve in a health system, conceptualize trust and reflect on its importance within the health system, and finally note the role of senior leaders and managers in supporting trust and responsiveness for co-designed innovation and change.

**Findings:**

Healthcare needs leaders and managers to embrace co-design that drives innovation now and in the future through people – leading to better healthcare for society at large. As authors we argue that it is now the time to shift our focus on the role of senior managers and leaders to embed co-design into health and social care structures, through creating and nurturing a culture of trust.

**Originality/value:**

Building public trust in the health system and interpersonal trust within the health system is an ongoing process that relies upon personal behavior of managers and senior leaders, organizational practices within the system, as well as political processes that underpin these practices. By implementing managerial, leadership and individual practices on all levels, senior managers and leaders provide a mechanism to increase both trust and responsiveness for co-design that supports innovation and change in the health system.

## Introduction

Co-design is value-based approach that brings diverse people together to build, refine or change parts of the health and social care system (Ní Shé and Harrison, 2021). This approach moves from consulting to enabling the involvement of all from the start (Ní Shé *et al.*, 2019; Robert *et al.*, 2022). The process ensures and supports all relevant partners to be involved in defining the problem, designing the solution and monitoring and championing the implementation. By gathering all key stakeholders, including those with lived experiences, co-design encourages the formation of equal and reciprocal relationships underpinned by trust and open communication. Co-design should not include a one-off workshop but focus on long-term evolving partnership that drives innovation and enables sustainable change within a specific context (O’Donnell *et al.*, 2019; Ní Shé and Harrison, 2021; Busch and Palmås, 2023). There is a concern that co-design is the latest part of “managerial fads and fashion’ (Abrahamson, 1991). The recent literature has started to reflect on this with literature on a focus on potential corruption and unintended consequences of co-design (Ní Shé and Harrison, 2021; Busch and Palmås, 2023)

As Evans and Terrey (2016, p. 243) outline co-design done badly can “destroy trust systems”; but when done well, it can help “solve policy and delivery problems, stabilise turbulent lives, and improve life chances”.

A co-design approach provides a mechanism to move beyond stumbling blocks because it is based on diverse inclusion of members enabled by trust and context specific solutions (O’Donnell *et al.*, 2019, 2022). Meaningful and inclusive involvement of all relevant partners in the design, management, and implementation of change requires skills, time, flexibility, and resources (Ní Shé *et al.*, 2019; Locock *et al.*, 2022). However, challenges exist. Post the COVID 19-pandemic change fatigue has emerged as staff burnout increased and trust in their organization to respond to the change decreased (Morain and Peter, 2023). The literature also outlines that involvement requires responsiveness, resourcing and support from senior leaders and managers to ensure the change is sustained (Boaz *et al.*, 2016; Harrison *et al*., 2022b).

This viewpoint article is exploratory developed form our discussions, interpretations and experiences of co-design. We explore the role of leaders and mangers in enhancing trust in their organizations by responding and enabling co-design innovation and change. Current co-design literature remains scant on interactions between senior leaders and mangers, healthcare staff and service users in developing a culture of trust to support co-designed innovation and change. As authors’ we argue that it is now time to shift the academic focus on the role of senior managers and leaders to embed co-design into health and social care structures. The literature stressed that in order to enable participatory governance approaches such as co-design there is a need to focus on the role of senior leaders and managers (Fung, 2015; Ní Shé *et al.*, 2020b; Harrison *et al*., 2022b). We outline what co-design innovation and change involves in a health system, reflect, and understand what trust is and finally note the role of senior leaders and managers supporting trust and responsiveness for co-designed innovation and change.

## Co-design innovation and change in health systems

Central to an adaptive health system is the ability to be responsive to change that is inclusive of the relevant “actors” and addresses their needs within the local context of delivery (Rapport *et al.*, 2022). It is well recognized that undertaking change within health systems is challenging. Much of the recent evidence notes that “change failure” is ever-present (Harrison *et al*., 2022a, b). It can often be poorly managed and lacks inclusive involvement or support from senior leaders. More recently, as levels of burnout increase across the health and social care system, staff' experience of creating change and enabling change is deemed limited (Creese *et al.*, 2021; Harrison *et al*., 2022a). This is further exemplified by the significant psychological and physiological impact of burnout resulting in higher staff turnover, absenteeism and presenteeism within healthcare organizations (Kearney *et al.*, 2020) making it difficult to successfully co-design innovate and change within such a challenging context (Fulham-McQuillan *et al.*, 2023; Byrne *et al.*, 2023).

However, such challenges can be managed effectively with the right leadership and management to embrace co-design and drive innovation and change. To effectively achieve this leaders and managers need to demonstrate “authenticity, openness, humility, compassion, and appreciation, where everyone has a voice” (Kearney, 2022, p. 156) and ensure co-design and innovation is supported at all levels. This support can include embracing co-design that drives innovation and positive change, providing necessary resources and time, as well as embedding co-design and innovation into the organizational culture.

Innovation does not happen without people and the most innovative staff are generally not at the senior level of the organization (Kearney, 2021). Frontline staff play a significant role given their daily experience of the system and must be supported to fully utilize their creativity (Kearney, 2022). Leaders and managers that embed co-design and innovation as part of the culture and support staff at all levels need to utilize their core competencies and work together with key stakeholders. In doing so, they think beyond the current boundaries that are driving the development of innovations, to advance scientific knowledge and address patient needs now and in the future.

Co-design is of significant value to health systems in driving innovation and addressing patient needs. This in turn can save lives as well and improve the quality of life and patient care. Co-design and innovation in healthcare is not an option but a necessity to address the significant and potentially unprecedented challenges facing today’s healthcare organizations (Kearney, 2022). For this to be successfully achieved both leaders and managers must engender trust and support staff to embrace co-design, drive innovation and change. This requires breaking the current status quo as we cannot continue to do the same thing and expect different results.

## Understanding trust

Trust is argued to be the most fundamental relationship attribute that affects behaviors and outcomes within the health system (Hall *et al.*, 2001). Trust can be conceptualized as an acceptance of the uncertainty and the risk associated with the expectation that the other party will act to the best of their ability and in good faith (Sheehan *et al.*, 2020). It is a psychological state that captures an optimistic disposition of an individual and a level of confidence in the moral orientation of fellow citizens (Li *et al.*, 2018), which denotes both the affective and ethical components of the concept. Trusting involves a decision to willingly give away power and thus make oneself vulnerable to the actions of another party, and it is predicated on the possibility of a negative outcome for “if there is no possibility of betrayal, then we are not talking about trust” (Sucher and Gupta, 2021). Trust and vulnerability are therefore intertwined as the process of trusting creates vulnerability, and yet trust arises from conditions of vulnerability that are unavoidable in medicine (Hall *et al.*, 2001).

There is an agreement in the literature around the key features that constitute trust. Trust is thought to be a future-oriented concept for it is based upon an expectation of how another party will behave in the future (Gilson, 2003). It is also context specific as structural elements such as institutional barriers, norms, and values affect one’s ability to trust (Gilfoyle *et al.*, 2022) as do professional norms and power dynamics inherent in healthcare organizations (Gilbert, 2005). Thirdly, trust is a relational concept as it involves at least two parties and a task, notwithstanding differences between trusting an individual or an institution, which amplifies complexities around the behavior (Sheehan *et al.*, 2020). Furthermore, interpersonal and impersonal trust signifies trust in an individual, and trust based on roles, systems or reputation respectively (Atkinson and Butcher, 2003). Given the multifaceted nature of trust, Robin *et al.* (2020) propose the concept “complex ecologies of trust” that posit the interplay between trust, distrust, skepticism, and their dialectic combination in the center of inquiry. In organizations, trust creates higher levels of workplace cooperation and positive performance outcomes and enables sustainable partnerships and social networks (Gilfoyle *et al.*, 2022). A meta-review of 112 independent studies confirmed that trust is positively related to team performance (De Jong *et al.*, 2016), and therefore creates the conditions for solving collective action problems (Uslaner, 2002). The effects of trust and created social value closely align with the motivations underpinning co-design approaches (Ní Shé *et al.*, 2020a). However, the asymmetries in the trust-building and trust-destroying processes (Kramer, 1999) and a complex interplay between interpersonal relationships, organizational practices and political processes inherent in building trust in health systems (Gilson, 2003) render this process less straightforward. In this context, the role of senior leaders and managers in creating and nurturing the conditions for trust becomes pivotal.

## Senior leaders and managers supporting trust and responsiveness for co-designed change and innovation

Embedding co-design change within a complex organization remains a challenge (Harrison *et al*., 2022b). The term “to embed” is often used in health and social care aligned to change or long-term implementation of evidence-based initiatives. However, the word “embed” has not been clearly defined in the literature and is a complex concept. Shifting from one off initiatives to long term embeddedness requires reflections on changing structures, supporting staff and resourcing and monitoring co-designed change (Fagotto *et al.*, 2019). Chwalisz links embeddedness with becoming “a permanent part of the policy cycle” (Chwalisz, 2020 p. 121). Bussu and colleagues outline three dimensions of embeddedness as “temporal, spatial and practices” (Bussu *et al.*, 2022). Temporal embeddedness is focused on shifting from an “exception” to permanency within spaces where power and decision-making are operationalized. The third element of practices is aligning the correct resources, policy and mechanisms (Bussu *et al.*, 2022) and these practices can be utilized to enhance trust and support co-design for innovation and change.

**Figure 1:**
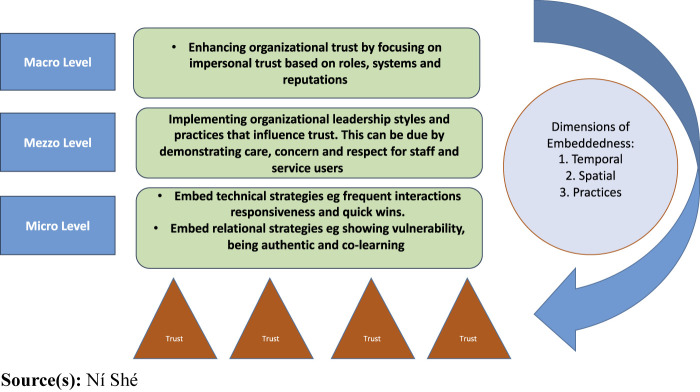
Examples of enabling trust mechanisms supporting co-design innovation

Senior leaders and mangers have an interest in creating the conditions of trust to reduce transaction costs (Kramer, 1999; Kramer and Cook, 2004), secure communication and dialogue (Gilson, 2003), and increase motivation, work engagement and the quality-of-service delivery. Organizational, managerial, leadership and individual practices that create and nurture trust, and thus support co-designed innovation and change, need to operate simultaneously on several levels. On a macro level, impersonal trust based on roles, systems or reputation of a healthcare institution can serve as a precursor and guarantor or interpersonal trust (Gilson, 2003; Atkinson and Butcher, 2003). The relationships between senior leaders and mangers, healthcare staff and service users are shaped by the institution in which they are embedded, and public trust in healthcare institutions may serve as a foundation of trust between these agents (Figure 1).

Figure 1: Senior leaders and managers supporting trust and responsiveness for co-designed change and innovation.

On a mezzo level, senior leaders and managers can support co-designed innovation and change by implementing organizational leadership styles and practices that influence trust. From a relationship-based perspective, the most important precursors of trust are transformational leadership, perceived organizational support and interactional justice (Dirks and Ferrin, 2002). By demonstrating individualized care, concern and respect for healthcare staff and service users, as well as support at every organizational level, managers and leaders can engender transformational leadership and nurture trust (Dirks and Ferrin, 2002). This process can further be aided through the implementation of high-trust management practices that encourage patient-centric and cooperative problem-solving approaches (Gilson, 2003).

Finally, on a micro level, leaders can adopt two core sets of strategies to build trusting high-quality relationships with the stakeholders and support effective implementation (Metz *et al.*, 2020, 2022). Technical strategies for developing trust include frequent interactions, responsiveness, demonstration of expertise, and achievement of quick wins, whereas relational strategies include showing vulnerability, being authentic, engaging in co-learning and empathy-driven exchanges, and adopting bi-directional communication. Taken together relational and technical strategies can help build trust with and among the stakeholders by demonstrating knowledge, reliability and competence, and strengthening the quality and reciprocity of relationships respectively. Similarly, relational signaling theory suggests that leaders can foster trust by performing actions that send signals to other stakeholders that they wish to maintain the relationships, and attend to needs to the other (Six *et al.*, 2010). This is achieved through recognizing the legitimacy of others’ needs and providing care and assistance, and preventing disappointment by clarifying expectations and surfacing and settling differences. In addition, behaviors that inspire trust rely upon the notion of a “moral manager”, which includes role modeling ethical behavior through visible action, and communicating about ethics and values to foster a culture of open dialogue (Treviño *et al.*, 2000). The level of consistency between desired and observed ethical behavior of leaders, as perceived by followers, is therefore a predictor of trust (Van den Akker *et al.*, 2009). Finally, senior leaders and managers need to secure funding arrangements and resource allocation to build trust with service users (Gilson, 2003), and engage in a reflective practice to interrogate their attitudes towards vulnerable social groups for trust appears to favor the privileged (Li *et al.*, 2018, Baroudi *et al.*, 2022).

## Conclusion

Building public trust in the healthcare system is an ongoing process that relies upon personal behavior of managers and senior leaders, organizational practices within the healthcare system, as well as political processes that underpin these practices (Gilson, 2003). Health systems need leaders and managers to support innovation and change. A co-design approach requires reimagining the governance and processes models to enable shared power (Harrison *et al.*, 2021; Busch and Palmås, 2023). For example, consideration must be given to how to involve seldom included groups who as people may experience barriers in participation in healthcare decision-making or accessing healthcare services (Islam *et al.*, 2021; Ní Shé *et al.*, 2019). A default workshop held in a hospital room presented as co-design is not sufficient as it may cause more harm than good with those involved if no change occurs (Locock *et al.*, 2022).

Existing organizational structures and practices may not provide an opportunity for equal involvement with seldom included and hard-to-reach social groups. Understanding these challenges and proving an enabling culture is critical for leaders and mangers (Busch and Palmås, 2023). We believe that building and nurturing a culture of trust on all levels within but also transcending the health system is a core element for enabling successful co-design for innovation and change. This viewpoint advances on the gaps in the literature to stress the critical role of senior leaders and mangers in creating the conditions of trust and being responsive to what has been co-designed to enable innovation and change. We recognize that further empirical work is required to build the evidence base. Health systems needs leaders and managers to embrace co-design that drives innovation now and in the future through people – leading to better healthcare for society at large.

## References

[ref001] Abrahamson, E. (1991), “Managerial fads and fashions: the diffusion and rejection of innovations”, Academy of Management Review, Vol. 16 No. 3, pp. 586-612, doi: 10.5465/amr.1991.4279484.

[ref002] Atkinson, S. and Butcher, D. (2003), “Trust in managerial relationships”, Journal of Managerial Psychology, Vol. 18 No. 4, pp. 282-304, doi: 10.1108/02683940310473064.

[ref050] Baroudi, M., Goicolea, I., Hurtig, A.K. and San-Sebastian, M. (2022), “Social factors associated with trust in the health system in northern Sweden: a cross-sectional study”, BMC Public Health, Vol. 22 No. 1, p. 881, doi: 10.1186/s12889-022-13332-4. 35509072 PMC9065232

[ref003] Boaz, A., Robert, G., Locock, L., Gordon, S., Gager, M., Vougioukalou, S., Ziebland, S. and Fielden, J. (2016), “What patients do and their impact on implementation: an ethnographic study of participatory quality improvement projects in English acute hospitals. Edited by aoife M. McDermott and anne reff pedersen”, Journal of Health Organization and Management, Vol. 30 No. 2, pp. 258-278, doi: 10.1108/JHOM-02-2015-0027. 27052625

[ref004] Busch, O.V. and Palmås, K. (2023), The Corruption of Co-design: Political and Social Conflicts in Participatory Design Thinking, Taylor & Francis, Abingdon-on-Thames.

[ref005] Bussu, S., Bua, A., Dean, R. and Smith, G. (2022), “Embedding participatory governance”, Critical Policy Studies, pp. 1-13, doi: 10.1080/19460171.2022.2053179.

[ref006] Byrne, J.-P., Humphries, N., McMurray, R. and Scotter, C. (2023), “COVID-19 and healthcare worker mental well-being: comparative case studies on interventions in Six countries”, Health Policy, Vol. 135, 104863, doi: 10.1016/j.healthpol.2023.104863. 37399678 PMC10292916

[ref007] Chwalisz, C. (2020), Reimagining Democratic Institutions: Why and How to Embed Public Deliberation, OECD, Paris, doi: 10.1787/056573fa-en.

[ref008] Creese, J., Byrne, J.-P., Matthews, A., McDermott, A.M., Conway, E. and Humphries, N. (2021), “‘I feel I have No voice’: hospital doctors' workplace silence in Ireland”, Journal of Health Organization and Management, Vol. 35 No. 9, pp. 178-194, doi: 10.1108/JHOM-08-2020-0353. PMC9136865 33955211

[ref009] De Jong, B.A., Dirks, K.T. and Gillespie, N. (2016), “Trust and team performance: a meta-analysis of main effects, moderators, and covariates”, The Journal of Applied Psychology, Vol. 101 No. 8, pp. 1134-1150, doi: 10.1037/apl0000110. 27123697

[ref047] Dirks, K.T. and Ferrin, D.L. (2002), “Trust in leadership: meta-analytic findings and implications for research and practice”, Journal of Applied Psychology, Vol. 87 No. 4, pp. 611-628, doi: 10.1037/0021-9010.87.4.611. 12184567

[ref010] Evans, M. and Terrey, N. (2016), “Co-design with citizens and stakeholders”, in Stoker, G. and Evans, M. (Eds), Evidence-Based Policy Making in the Social Sciences: Methods that Matter (Bristol, Policy Press Scholarship), pp. 243-262.

[ref011] Fagotto, E., Burgués, V.A. and Fung, A. (2019), “A taxonomy to engage patients: objectives, design, and patient activation”, NEJM Catalyst, August, available at: https://catalyst.nejm.org/doi/full/10.1056/CAT.19.0626

[ref012] Fulham-McQuillan, H., O'Donovan, R., Buckley, C.M., Crowley, P., Gilmore, B., Martin, J., McAuliffe, E., Martin, G., Moore, G., Morrissey, M., Nicholson, E., Shé, É.N., O'Hara, M.C., Segurado, R., Sweeney, M.R., Wall, P. and De Brún, A. (2023), “Exploring the psychological impact of contact tracing work on staff during the COVID-19 pandemic”, BMC Health Services Research, Vol. 23 No. 1, 602, doi: 10.1186/s12913-023-09566-6. 37291553 PMC10250071

[ref013] Fung, A. (2015), “Putting the public back into governance: the challenges of citizen participation and its future”, Public Administration Review, Vol. 75 No. 4, pp. 513-522, doi: 10.1111/puar.12361.

[ref014] Gilbert, T. (2005), “Impersonal trust and professional authority: exploring the dynamics”, available at: https://onlinelibrary.wiley.com/doi/full/10.1111/j.1365-2648.2004.03332.x 10.1111/j.1365-2648.2004.03332.x 15737217

[ref015] Gilfoyle, M., MacFarlane, A. and Salsberg, J. (2022), “Conceptualising, operationalising, and measuring trust in participatory health research networks: a scoping review”, Systematic Reviews, Vol. 11 No. 1, 40, doi: 10.1186/s13643-022-01910-x. 35249553 PMC8900447

[ref016] Gilson, L. (2003), “Trust and the development of health care as a social institution”, Social Science and Medicine, Vol. 56 No. 7, pp. 1453-1468, doi: 10.1016/s0277-9536(02)00142-9. 12614697

[ref017] Hall, M.A., Dugan, E., Zheng, B. and Mishra, A.K. (2001), “Trust in physicians and medical institutions: what is it, can it Be measured, and does it matter?”, The Milbank Quarterly, Vol. 79 No. 4, pp. 613-639, doi: 10.1111/1468-0009.00223. 11789119 PMC2751209

[ref018] Harrison, R., Chin, M., Ni She, E., Harrison, R., Chin, M. and Ni She, E. (2021), “What does Co-design mean for Australia's diverse clinical workforce?”, Australian Health Review, Vol. 46 No. 1, pp. 60-61, doi: 10.1071/AH21116. 34454639

[ref019] Harrison, R., Chauhan, A., Le-Dao, H., Minbashian, A., Ramesh, W., Fischer, S. and Schwarz, G. (2022a), “Achieving change readiness for health service innovations”, Nursing Forum, Vol. 57 No. 4, pp. 603-607, doi: 10.1111/nuf.12713. 35182394 PMC9545616

[ref020] Harrison, R., Chauhan, A., Minbashian, A., Ryan, M.M. and Schwarz, G. (2022b), “Is gaining affective commitment the missing strategy for successful change management in healthcare?”, Journal of Healthcare Leadership, Vol. 14, pp. 1-4, doi: 10.2147/JHL.S347987. 35082547 PMC8784667

[ref021] Islam, S., Joseph, O., Chaudry, A., Forde, D., Keane, A., Wilson, C., Begum, N., Parsons, S., Grey, T., Holmes, L. and Starling, B. (2021), “We are not hard to reach, but we may find it hard to trust’ …. Involving and engaging ‘seldom listened to’ community voices in clinical translational health research: a social innovation approach”, Research Involvement and Engagement, Vol. 7 No. 1, 46, doi: 10.1186/s40900-021-00292-z. 34174961 PMC8234650

[ref022] Kearney, C. (2021), “Leading innovation in healthcare in unprecedented times”, Health Manager, (blog), available at: https://healthmanager.ie/2021/12/leading-innovation-in-healthcare-in-unprecedented-times/ (accessed 6 December 2021).

[ref023] Kearney, C. (2022), Leading Innovation and Entrepreneurship in Healthcare: A Global Perspective, Edward Elgar Publishing, Cheltenham, available at: http://ebookcentral.proquest.com/lib/rcsidublin/detail.action?docID=6869395

[ref024] Kearney, C., Dunne, P. and Wales, W.J. (2020), “Entrepreneurial orientation and burnout among healthcare professionals”, Journal of Health Organization and Management, Vol. 34 No. 1, pp. 16-22, doi: 10.1108/JHOM-09-2019-0259. 31994848

[ref045] Kramer, R.M. (1999), “Trust and distrust in organizations: emerging perspectives, enduring questions”, Annual Review of Psychology, Vol. 50, pp. 569-598, doi: 10.1146/annurev.psych.50.1.569. 15012464

[ref046] Kramer, R.M. and Cook, K.S. (Eds) (2004), Trust and Distrust in Organizations: Dilemmas and Approaches, Russell Sage Foundation.

[ref025] Li, Y., Smith, N. and Dangerfield, P. (2018), “Social trust: the impact of social networks and inequality”, British Social Attitudes, Vol. 35, pp. 1-25, available at: https://research.manchester.ac.uk/en/publications/social-trust-the-impact-of-social-networks-and-inequality

[ref026] Locock, L., O'Donnell, D., Donnelly, S., Ellis, L., Kroll, T., Shé, É.Ní and Ryan, S. (2022), “‘Language has been granted too much Power’.1,p.1 challenging the power of words with time and flexibility in the precommencement stage of research involving those with cognitive impairment”, Health Expectations, Vol. 25 No. 6, pp. 2609-2613, doi: 10.1111/hex.13576. 36097364 PMC9700140

[ref027] Metz, A., Annette, B., Todd, J., Farley, A. and Leah, B. (2020), “Are relationships as important as strategies for successful implementation of evidence-informed programs and practices? [Blog] Transforming Evidence”, available at: https://transforming-evidence.org/blog/importance-of-relationships-is-under-recognised-by-research-into-evidence-implementation (accessed 12 December 2023).

[ref028] Metz, A., Todd, J., Farley, A., Annette, B., Leah, B. and Melissa, V. (2022), “Building trusting relationships to support implementation: a proposed theoretical model”, Frontier Health Service, Vol. 2, 894599, doi: 10.3389/frhs.2022.894599. PMC10012819 36925800

[ref029] Morain, C.O. and Peter, A. (2023), “Employees are losing patience with change initiatives”, Harvard Business Review, available at: https://hbr.org/2023/05/employees-are-losing-patience-with-change-initiatives (accessed 9 May 2023).

[ref037] Ní Shé, É., Cassidy, J., Carmel, D., Aoife, D.B., Sarah, D., Emma, D., Nikki, D., Foley, M., Galvin, M., Harkin, M., Killilea, M., Kroll, T., Lacey, V., Lambert, V., McLoughlin, S., Mitchell, D., Murphy, E., Mwendwa, P., Nicholson, E., O'Donnell, D. and O'Philbin, L. (2020a), “Minding the gap: identifying values to enable public and patient involvement at the pre-commencement stage of research projects”, Research Involvement and Engagement, Vol. 6 No. 1, 46, doi: 10.1186/s40900-020-00220-7. 32765898 PMC7396939

[ref035] Ní Shé, É. and Harrison, R. (2021), “Mitigating unintended consequences of Co-design in health care”, Health Expectations, Vol. 24 No. 5, pp. 1551-1556, doi: 10.1111/hex.13308. 34339528 PMC8483209

[ref036] Ní Shé, É., Morton, S., Lambert, V., Cheallaigh, C.N., Lacey, V., Dunn, E., Loughnane, C., McCann, A., Adshead, M. and Kroll, T. (2019), “Clarifying the mechanisms and resources that enable the reciprocal involvement of seldom heard groups in health and social care research: a collaborative rapid realist review process”, Health Expectations : An International Journal of Public Participation in Health Care and Health Policy, Vol. 22 No. 3, pp. 298-306, doi: 10.1111/hex.12865. 30729621 PMC6543157

[ref038] Ní Shé, É., O'Donnell, D., Donnelly, S., Davies, C., Fattori, F. and Kroll, T. (2020b), “What bothers me most is the disparity between the choices that people have or don't have’: a qualitative study on the health systems responsiveness to implementing the assisted decision-making (capacity) act in Ireland”, International Journal of Environmental Research and Public Health, Vol. 17 No. 9, 3294, doi: 10.3390/ijerph17093294. 32397345 PMC7246817

[ref030] O'Donnell, D., Ní Shé, É., McCarthy, M., Thornton, S., Doran, T., Smith, F., Barry, O.'B., Milton, J., Savin, B., Donnellan, A., Callan, E., McAuliffe, E., Gray, S., Carey, T., Boyle, N., O'Brien, M., Patton, A., Bailey, J., O'Shea, D. and Cooney Marie, T. (2019), “Enabling public, patient and practitioner involvement in Co-designing frailty pathways in the acute care setting”, BMC Health Services Research, Vol. 19 No. 1, 797, doi: 10.1186/s12913-019-4626-8. 31690304 PMC6833297

[ref031] O'Donnell, D., O'Donoghue, G., Ní Shé, É., O'Shea, M. and Donnelly, S. (2022), “Developing competence in interprofessional collaboration within integrated care teams for older people in the republic of Ireland: a starter kit”, Journal of Interprofessional Care, Vol. 37 No. 3, pp. 480-490, doi: 10.1080/13561820.2022.2075332. 35880753

[ref032] Rapport, F., Smith, J., Hutchinson, K., Clay-Williams, R., Churruca, K., Bierbaum, M. and Braithwaite, J. (2022), “Too much theory and not enough practice? The challenge of implementation science application in healthcare practice”, Journal of Evaluation in Clinical Practice, Vol. 28 No. 6, pp. 991-1002, doi: 10.1111/jep.13600. 34268832

[ref033] Robert, G., Locock, L., Williams, O., Cornwell, J., Donetto, S. and Goodrich, J. (2022), “Co-Producing and Co-Designing”, Elements of Improving Quality and Safety in Healthcare, Cambridge University Press, Cambridge, doi: 10.1017/9781009237024.

[ref034] Robin, S., Kennedy, H. and Jones, R. (2020), “'Complex ecologies of trust in data practices and data-driven systems, Information”, Communication and Society, Vol. 23 No. 6, pp. 817-832, doi: 10.1080/1369118X.2020.1748090.

[ref039] Sheehan, M., Friesen, P., Balmer, A., Cheeks, C., Davidson, S., James, D., Douglas, F., Keats-Rohan, K., Lawrence, R. and Shafiq, K. (2020), “Trust, trustworthiness and sharing patient data for research”, Journal of Medical Ethics, Vol. 47 No. 12, e26, doi: 10.1136/medethics-2019-106048. 32424061

[ref040] Six, F., Bart, N. and Adriaan, H. (2010), “Actions that build interpersonal trust: a relational signalling perspective”, Review of Social Economy, Vol. 68 No. 3, pp. 285-315, doi: 10.1080/00346760902756487.

[ref041] Sucher, S. and Gupta, S. (2021), “The power of trust: how companies build it, lose it, regain it - book - faculty & research - harvard business school”, available at: https://www.hbs.edu/faculty/Pages/item.aspx?num=59637

[ref048] Treviño, L.K., Hartman, L.P. and Brown, M. (2000), “Moral person and moral manager: how executives develop a reputation for ethical leadership”, California Management Review, Vol. 42 No. 4, pp. 128-142, doi: 10.2307/41166057.

[ref042] Uslaner, E.M. (2002), The Moral Foundations of Trust, Cambridge University Press, Cambridge, doi: 10.1017/CBO9780511614934.

[ref049] Van den Akker, L., Heres, L., Lasthuizen, K.M. and Six, F.E. (2009), “Ethical leadership and trust: it’s all about meeting expectations”, International Journal of Leadership Studies, Vol. 5 No. 2, pp. 102-122.

